# High-Efficiency Precision Polishing Using Fiber Brush–Shear-Thickening Fluid Composites

**DOI:** 10.3390/mi15121497

**Published:** 2024-12-15

**Authors:** Zepeng Gong, Yaodong Jin, Qianqian Cao, Xiaoxing Dong, Yongjie Shi, Fengli Huang, Lujuan Li, Zhongyu Piao

**Affiliations:** 1 College of Mechanical Engineering, Zhejiang University of Technology, Hangzhou 310023, China; 221122020442@zjut.edu.cn (Z.G.); zypiao@zjut.edu.cn (Z.P.); 2 College of Information Science and Engineering, Jiaxing University, Jiaxing 314001, China; li.lujuan@foxmail.com; 3 School of Physical Science and Technology, Lanzhou University, Lanzhou 730000, China; 320220935471@lzu.edu.cn; 4 School of Intelligent Manufacturing, Jiaxing Vocational Technical College, Jiaxing 314036, China; jiesy2007@126.com

**Keywords:** shear-thickening fluid, rheology, precision processing, fiber brush

## Abstract

Shear-thickening fluid (STF) is widely applied in various practical engineering fields due to its rheological properties of increased viscosity under load. We investigated the integration of STF with fiber brushes to prepare a novel composite material for polishing applications. The impact of composite material properties is studied in surface finish, specifically roughness and morphology, across flat and uneven surfaces. The effects of the critical variables, including polishing speed, feed depth, and STF concentration, are analyzed through experimentation and simulation. After the STF polishing, the surface roughness of the aluminum alloy sample decreases from 3.125 μm to 0.528 μm, which increases the processing efficiency by 40% compared to Newton polishing slurry. The unique shear-thickening performance of the composite material ensures excellent surface quality and high efficiency in the precision machining of workpieces.

## 1. Introduction

Shear-thickening fluid (STF) as a type of intelligent material has garnered significant interest in the academic community due to its distinctive rheological characteristics [1,2,3]. STF represents a dispersed system composed of a dispersed phase suspended within a medium, which is relatively straightforward to prepare [4]. For example, polyethylene glycol and nanosilica mixed in a certain ratio can produce a shear-thickening effect. Freundlich et al. first found that STF viscosity increases sharply with shear rate [5]. This shear-thickening effect is caused by interactions between polymer chains under pressure to form a three-dimensional network that reduces fluidity and thus increases viscosity [6,7]. A ubiquitous blend of starch and water possesses intriguing STF properties: it remains smooth liquid when agitated gently, yet transits rapidly into thicker fluid when stirred vigorously [8,9,10]. STF has a wide range of applications, from STF-treated fabrics for enhanced puncture resistance to STF-filled straps in sports eyewear for improved impact protection [11,12]. In sports equipment such as tennis rackets, STF integration reduces the impact of bending and increases strength [13,14]. Wei et al. designed and fabricated STF-treated fabrics. They found that the force peak of the STF-treated fabrics in the puncture test is significantly increased compared to the untreated fabrics [15]. Morris et al. designed a system to replace the traditional belt of sports eyewear with a tubular belt filled with STF, which improves the protection of Personal Protective Equipment under the condition of impact [16]. The versatility and potential of STF make it an invaluable material for a variety of engineering applications.

Polishing is an important way to achieve a brighter, cleaner, smoother finish through smoothing the surface of a workpiece based on mechanical, ultrasonic, or electrolytic methods [17]. Traditional polishing techniques employing rigid tools frequently create imperfections, scratches, and micro-cracks, particularly when applied to intricate surface geometries. These limitations originate from the inherent challenges faced by rigid tools in adapting to uniformly treating complex shapes, which ultimately compromises the surface quality and integrity of the workpiece [18,19,20]. Flexible machining based on a multi-degree-of-freedom system and elasticity that avoids hard contact enables the flexible polishing of complex surfaces and reduces surface damage [21,22]. Zhang et al. implemented a magnetic float polishing process in which magnetic fluid and abrasives are arranged around a workpiece [23]. By applying a magnetic field, non-destructive polishing was achieved under the rotation of the spindle to achieve a roughness of 30 nm. Zhao et al. polished sapphire by mixing hard diamond and silicon diamond abrasives [24]. Their results showed that the removal rate obtained with the mixed abrasives can reach 0.58 nm/min, which is 52.6% higher than that of pure diamond abrasives, thus illustrating the role of abrasives in material removal. Zhang et al. enhanced polishing capability by coating SiC abrasive particles with Fe_3_O_4_ and leveraging the response of magnetic materials to magnetic fields [25]. Fiber brush polishing is a common flexible polishing method that is widely used for surface cleaning, polishing, and passivation through the combination of brushes and polishing solutions [26,27]. Roure et al. used flexible fibers coupled with a polishing solution for machining complex edges of carbide inserts [28]. Shao et al. introduced flexible fibers in cutting edge preparation, which effectively eliminates micro-defects and improves surface smoothness [29]. The soft variability of fiber brushes makes their adaptability good on complex surfaces, but their processing efficiency is low. According to the Preston equation, material removal rate (MRR) is a function of pressure and relative speed [30]. However, the rotation of flexible fiber brushes can only provide speed support and cannot offer enough holding power for the abrasive grains in the polishing solution, which is the root cause of low efficiency for brush polishing [31]. If the holding power of the abrasive grains can be advanced in the polishing process of the brush fiber, then the processing efficiency will be significantly improved. STF can be employed to effectively enhance holding power because its viscosity rises sharply at a certain speed and the shear force increases by several orders of magnitude [32]. 

Compared to the existing shear-thickening polishing methods, we use fiber brushes to assist in shear-thickening fluid polishing. As fiber brushes are flexible materials, they solve the problem of rigid materials being unable to process complex surfaces. By harnessing the innovative substitution of conventional low-viscosity polishing fluids with STF, and synergistically integrating this fluid with a flexible fiber brush, it is plausible to envision a substantial enhancement in the material removal proficiency during brush polishing applications targeting arbitrary curved surfaces. Consequently, this study endeavors to prepare a high-performance polishing medium by incorporating silicon carbide abrasives into a shear-thickening slurry, thereby creating fiber-reinforced shear-thickening composites that leverage the fiber brush dynamic properties for optimized velocity and retention force. Furthermore, we introduce an STF-assisted brush fiber polishing technique which is tailored for different surface geometries. To delve into the polishing mechanism of this composite material, we examine the effects of various parameters, including polishing velocity (ω), feed depth (d), cassava starch concentration ($C_{m}$), and abrasive concentration ($C_{t}$), on surface roughness and morphology for plane, concave, and convex surfaces.

## 2. Experimental Details

### 2.1. Materials

The SiC abrasive particles used in the article were sourced from Dongguan Baite Grinding Materials Co., Ltd. (Dongguan, China). The material of the fiber brush was 1000 mesh SiC, with an outer diameter of 100 mm and an inner hole of 20 mm, sourced from Qianshan Zhiyuan Brush Manufacturing Co., Ltd. (Qianshan, China). The starch was sourced from Hubei Yucheng E-commerce Co., Ltd. (Jingmen, China); the content of amylose was 25%, the content of amylopectin was 75%, the density was 0.45 g/cm^3^, and the surface area ratio was 0.153 m^2^/g. In the preparation of shear-thickening fluid (STF), high-purity silicon carbide (SiC) particles with a defined grade and an average particle diameter of 13 μm were dispersed within a colloidal suspension medium crafted from an aqueous cassava starch blend. This dispersion process aims to ensure uniform particle distribution, which is important for the subsequent performance characteristics of the STF.

**Figure 1:**
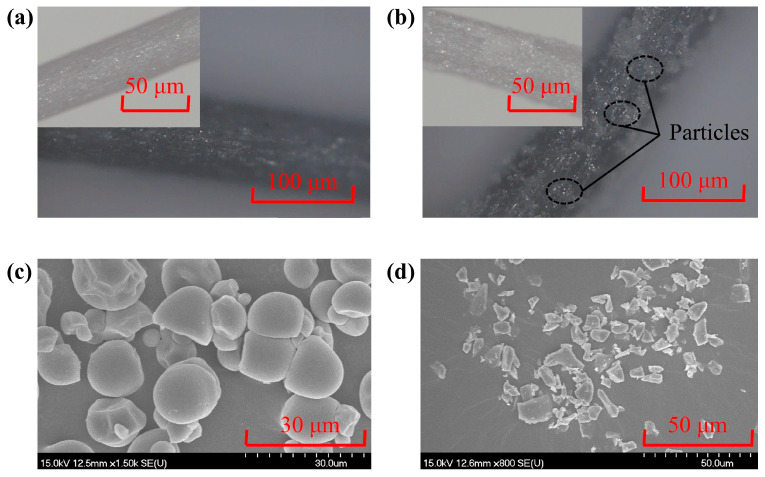
Micrographs and SEM images: (**a**) fiber brush, (**b**) fiber brush in the STF, (**c**) starch particles, and (**d**) SiC particles.

Figure 1 presents a microstructural analysis of the fiber brushes before and after the incorporation of the STF. Specifically, Figure 1a depicts the pristine state of the fiber bristles, namely their original morphology without any surface modification. Conversely, Figure 1b shows that after soak-drying by the STF; the interstices of the fiber bristles are interwoven with visible silicon carbide abrasive particles and cassava starch residues. The scanning electron microscopy (SEM) images in Figure 1c,d offer a magnified view of the individual cassava starch and SiC. The shear-thickening phenomenon occurs when they are mixed with deionized water in a certain ratio. The detailed data on the shear-thickening rheological characteristics will be shown in the next section.

### 2.2. Preparation of STF

In the STF, the cassava starch served as the primary thickening agent, which was dissolved and agitated in deionized water to create a homogeneous substrate medium. Subsequently, the SiC particles as the abrasive were thoroughly incorporated into the substrate fluid through rigorous stirring for 30 min to ensure a uniform dispersion of the STF mixture.

**Figure 2:**
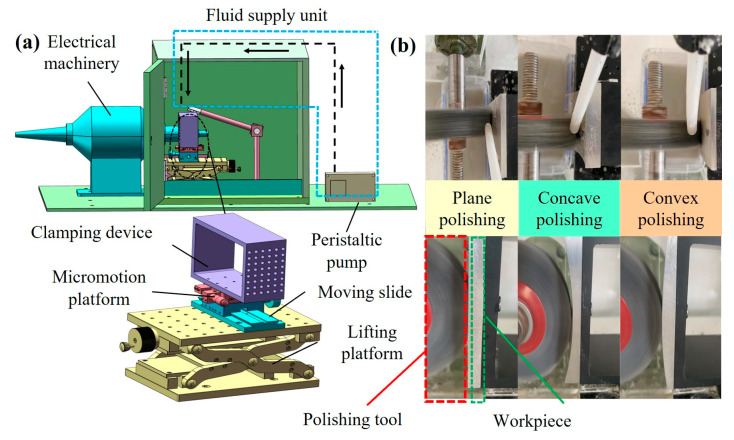
(**a**) Schematic diagrams of the experimental setup, and (**b**) different surface polishing states.

**Figure 3:**
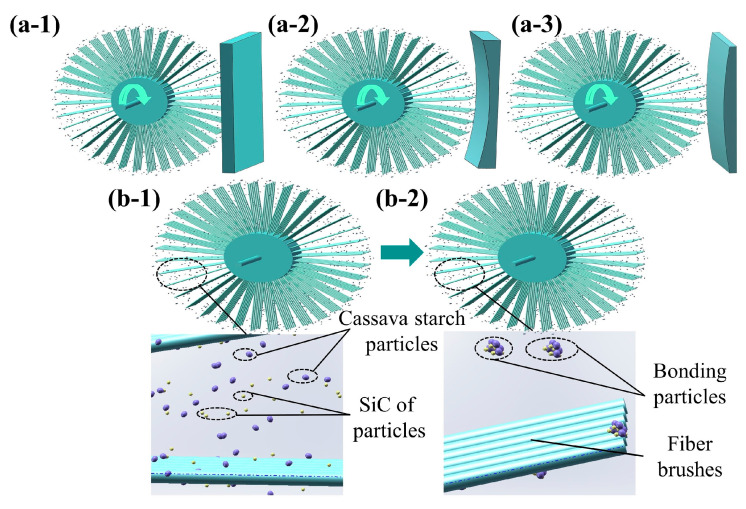
(**a**) Schematic diagrams of polishing for different surface structures, (**a-1**) plane polishing, (**a-2**) concave polishing, (**a-3**) convex polishing, and (**b**) state changes in the particles in the STF before and after polishing, (**b-1**) particles state without shear thickening, (**b-2**) particles state with shear thickening.

To examine the unique characteristics of the composite material, an experimental configuration was devised as shown in Figure 2. This setup integrates a motor for precise speed control, a lifting platform for the vertical adjustment of the workpiece, and a sliding mechanism for modulating the gap between the workpiece and the fiber brush. By employing a peristaltic pump, the STF is dispensed onto the fiber brush, where abrasive particles are embedded to facilitate the surface treatment of the workpiece. Figure 3a illustrates the schematic layout which is tailored for the surfaces with different curvatures. The interface between the workpiece and the fiber brush has a substantial velocity gradient, which can trigger the shear-thickening behavior. Consequently, this viscosity surge causes substantial hydrodynamic pressure ($P_{s}$), which translates into high normal force ($F_{n}$). The synergistic action of both shear and normal stresses enables the abrasive material to microscopically abrade the workpiece surface [33]. Figure 3b illustrates the interplay between starch particles and abrasive particles during polishing with fiber brushes. At low shear rates, these particles remain dispersed. However, as the shear rate exceeds a critical threshold, the colloidal particles within the STF rapidly aggregate, further forming particle clusters that contribute to their unique rheological properties [34]. These clusters subsequently hinder the fluidic motion within the system, leading to a pronounced shear-thickening effect. This is manifested macroscopically as an increase in the resistance to flow under applied shear stress [6].

## 3. Results and Discussion

### 3.1. Rheological Behavior of the STF

The rheological properties of the STF are intricately governed by the cassava starch concentration and slurry temperature [16]. To explore their relationship, a series of rheological tests were conducted by a TA DHR rotational rheometer equipped with a parallel plate with a diameter of 20 mm. Throughout the experiments, a consistent gap of 0.10 mm between the plates was maintained to ensure accuracy. The viscosity response of the STF to the shear rate was systematically analyzed under different cassava starch concentrations and slurry temperatures. To reduce measurement uncertainties and ensure the reliability of the obtained data, the steady-state viscosity assessments were replicated three times. The specific experimental parameters are detailed in Table 1.

**Figure 4:**
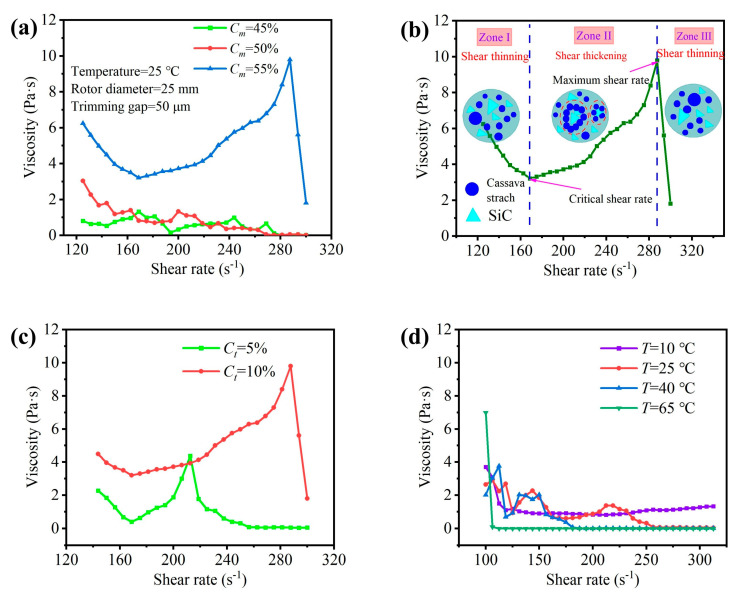
Fluid viscosity as a function of shear rate at (**a**) different cassava starch concentrations, (**c**) abrasive particle concentrations, and (**d**) temperature. (**b**) shows the different viscosity regimes of the STF ($C_{m}$ = 55%, $C_{t}$ = 10%, and T = 25 °C).

Figure 4a presents the comprehensive rheological properties of the STFs for different starch concentrations. An increase in the maximum viscosity of the STFs was observed with the increase in the starch concentration. The shear-thickening effect, triggered by an increase in the starch concentration, is responsible for the increase in the peak viscosity. At $C_{m}$ = 55%, the STF exhibits a distinct transition from shear-thinning to shear-thickening and finally back to shear-thinning (Figure 4b). At $C_{m}$ = 45% and 50%, the STF shows viscosity fluctuations. This deviates from the continuous shear-thinning–thickening–thinning pattern, suggesting that the dispersion with relatively low concentrations does not cause obvious shear-thickening behavior. At $C_{m}$ = 55% and $C_{t}$ = 10%, the STF can be divided into three distinct viscosity regimes in terms of the shear rate, designated as Zones I, II, and III [35]. The behavior of the STF in Zones I and III exhibits shear-thinning characteristics, where starch particles and abrasive particles are either freely suspended or subjected to excessive shear. Zone II represents the shear-thickening regime, characterized by a significant increase in viscosity that culminates at approximately 9.81 Pa·s at a shear rate of 288 s^−1^. This is consistent with the results in the literature [35]. The base liquid with $C_{m}$ = 55% for shear-thickening polishing was used to prepare a series of polishing liquids with different fractions of abrasive particles. The increase in abrasive particle concentration induces stronger inter-particle force and prompts particle aggregation in the dispersed phase. Under low shear rates, the abrasive particle concentration correlates positively with the initial viscosity of the polishing solutions, which underscores the influence of particle–particle interactions on the rheological properties. 

In Figure 4c, the increase in shear rate is accompanied by the higher peak viscosity of STFs prepared at high abrasive particle concentration, manifesting a pronounced shear-thickening effect. To investigate the influence of temperature on the rheological behavior of these fluids, we conducted experiments using the STF with SiC particles ($C_{t}$ = 5%). The rheological properties of the polishing liquid were measured at different temperatures: T = 10 °C, 25 °C, 40 °C, and 65 °C (Figure 4d). The change in temperature influences the Brownian motion of the particles in the STF and their dynamic behavior. In turn, it has a significant impact on the rheological behavior of the STF, which indicates the importance of temperature in controlling its flow behavior. At T = 25 °C and 40 °C, the polishing liquid exhibits distinct shear-thickening behavior. At T = 10 °C and 65 °C, the viscosity of the slurry rapidly decreases at the initial moment. At T = 10 °C, as the shear rate increases, the viscosity of the slurry decreases to around 1.081 Pa·s. At T = 65 °C, as the shear rate increases, the viscosity of the slurry decreases to around 0.012 Pa·s and remains stable. Because at low temperatures, the thermal motion rate of liquid molecules is slow, and the exchange and movement between molecules are not frequent, resulting in the high viscosity of the liquid during flow. At high temperatures, the thermal motion rate of liquid molecules accelerates, and the exchange and movement between molecules become more frequent, which makes the liquid flow more smoothly and reduces viscosity. Hence, the exact regulation of temperature in the STFs plays a pivotal role in optimizing the polishing process.

In this study, we also carried out numerical simulations to calculate the pressure distributions at the fiber/workpiece interface for different geometric surfaces. To ensure the simulation’s effectiveness, it is necessary to characterize the constitutive equation of STF. We used the power law for non-Newtonian fluids to describe the relationship between shear stress and shear rate:

(1) $$\tau =K\cdot |{\gamma |}^{n},$$

where τ is the shear stress, γ is the shear rate, K is the consistency coefficient, and n is the viscosity index. The viscosity of STF can be expressed as follows:

(2) $$\eta =K\cdot {\left|\gamma \right|}^{n-1},$$

**Figure 5:**
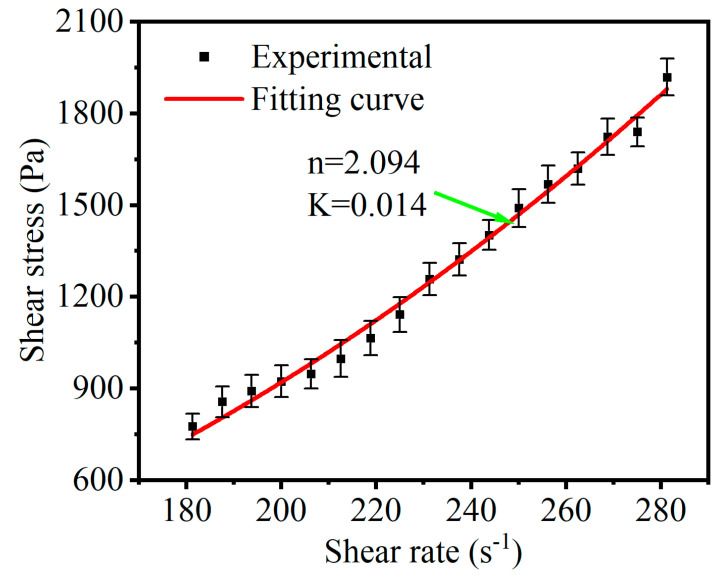
Shear stress via shear rate curve of STF ($C_{m}$ = 55%, $C_{t}$ = 10%, and T = 25 °C).

For the STF with $C_{m}$ = 55% and $C_{t}$ = 10%, the shear stress is plotted against the shear rate in Figure 5. It exhibits a behavior similar to the characteristics of the power law model of non-Newtonian fluids. Based on the robust Levenberg–Marquardt algorithm, the sampled data points are fitted to the power function outlined in Equation (1). Upon achieving convergence within the prescribed parameter bounds, the viscosity-related parameters are estimated as n= 2.094 and K= 0.01427, with a fitting correlation coefficient of 0.9996. This signifies an excellent agreement between the model and experimental observations. Consequently, the equations of the shear stress and viscosity can be expressed as follows:

(3) $$\tau =0.01427\cdot |{\gamma |}^{2.094},$$

(4) $$\eta =0.01427\cdot {\left|\gamma \right|}^{1.094},$$

### 3.2. Polishing Based on Fiber Brush–STF Composites

**Figure 6:**
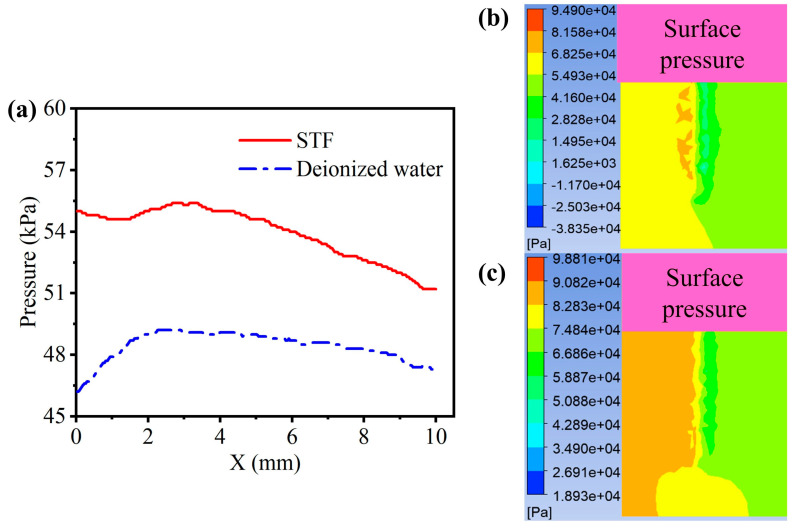
(**a**) Pressure curve along the centerline of the workpiece surface, and the pressure contour at the workpiece surface in (**b**) the deionized water and (**c**) the STF calculated through numerical simulations.

In the experiments, we investigated how the starch concentration, abrasive particle concentration, polishing speed, and feed depth influence the surface roughness of the workpiece both before and after the polishing process. To understand the mechanism of the fiber brush–STF polishing experiments, we first conducted numerical simulations of the polishing process based on the solid–liquid coupling methodology. Please refer to Appendix A for simulation details. Figure 6b and c show the distributions of the pressure field on the workpiece surface when the STF and deionized water are used as the polishing fluid, respectively. A higher pressure on the workpiece surface was observed for the STF polishing compared to the deionized water. Further, we calculated the pressure profile along the central line of the workpiece surface as shown in Figure 6a. The simulation results indicate that the surface pressure during the polishing process with the deionized water is approximately 0.049 MPa. In contrast, based on the STF polishing, the maximum pressure reaches roughly 0.056 MPa.

According to the Preston equation, *dz*/*dv* = *kvp*, dz/dv represents the material removal rate, k is the proportionality coefficient, p is the pressure of the abrasive on the workpiece, and v is the relative velocity between the abrasive and the workpiece [35], which posits a direct proportionality between the material removal rate and both the velocity and pressure at the polishing interface; the increase in surface pressure induced by the STF is in favor of enhancing material removal efficiency. Thus, STF polishing is a promising strategy for achieving high ultra-precise machining performance.

The STF in the experiments is integrated with the fiber brush through a peristaltic pump. The fiber brush is driven by a motor to make the STF achieve the shear-thickening effect, and thus the workpiece can be polished by abrasive particles under the STF with the shear-thickening property. The different surfaces (plane, concave, and convex) were polished for 30 min, and the value of surface roughness Ra was recorded every 5 min. We analyzed the effects of four factors on the polishing performance, namely cassava starch concentration, motor speed, feed distance, and silicon carbide concentration as shown in Table 2.

In the shear-thickening state, the viscosity of STF increases, making it easier for abrasive particles to be tightly wrapped by the fluid and transferred to the material surface, thereby improving the efficiency of material removal. The shear-thickening behavior causes the STF to generate higher surface pressure when subjected to shear force. This increased surface pressure, combined with frictional force, enables the abrasive particles to more effectively remove small protrusions and defects on the material surface. When the shear force increases, the viscosity rapidly increases, and when the shear force decreases, the viscosity rapidly decreases again, which enables the STF to automatically adjust its mechanical behavior according to the local conditions during the polishing process, thereby optimizing the polishing uniformity.

We used 6061 aluminum alloy as the test sample in all the experiments. To ensure consistent initial conditions, the surface roughness of each workpiece was ground to a range of 2.7 μm to 3.3 μm by precision grinding techniques. Subsequently, the surface roughness of the workpieces was rigorously assessed using a high-precision surface roughness tester. This assessment involves the measurement of two distinct pairs of vertically oriented lines on each workpiece, with the final Ra value determined as the arithmetic mean of four individual measurements. This methodology ensures accuracy and reproducibility in the quantification of surface roughness, serving as a robust basis for subsequent analyses.

**Figure 7:**
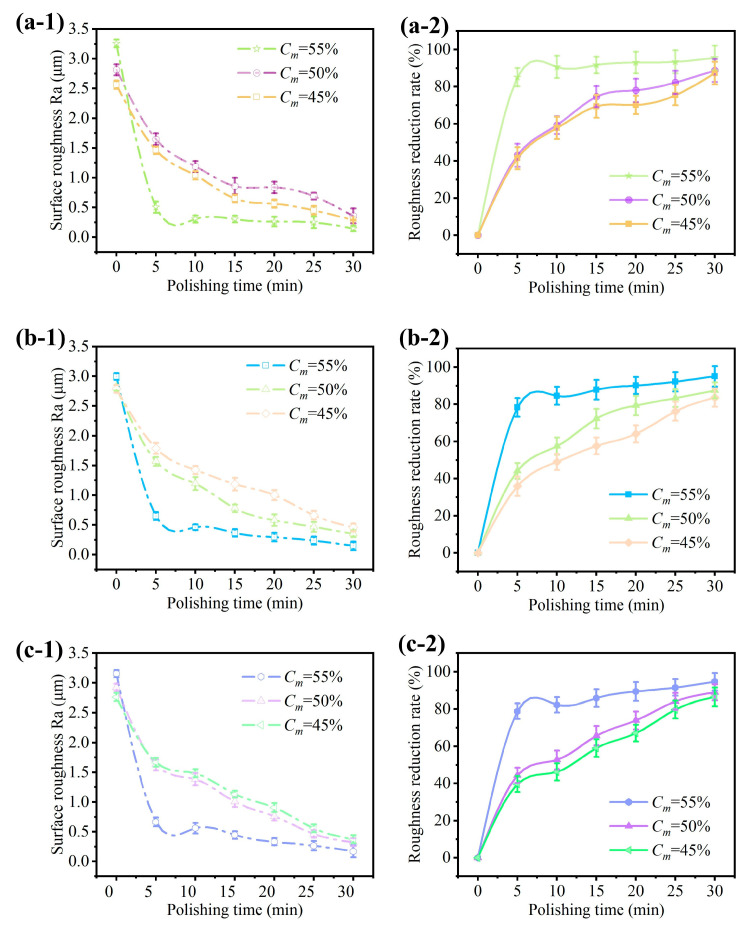
Influence of the starch concentration on the surface roughness and its reduction rate for different surface geometries: (**a**) plane surface, (**b**) concave surface, and (**c**) convex surface.

Figure 7 presents the roughness variation curves from the polishing on different surface geometries by using the STF solution at ω = 1000 rpm. The solution was prepared with $C_{t}$ = 10%, coupled with different concentrations of cassava starch at $C_{m}$ = 45%, 50%, and 55%. During the polishing process, the feed depth was set at d = 8 mm. The rheological tests in the previous section reveal that the shear-thickening behavior is observed at $C_{m}$ = 55%, while it does not occur at $C_{m}=$ 45% and 50%. It can be seen that the roughness of the polished surface decreases as the cassava starch concentration increases. The decrease rate in roughness is faster in the first 5 min of the polishing process. The roughness curves exhibit a declining trend. It indicates that the surface roughness is gradually improved during the polishing operation with the fiber brush. Due to the shear-thickening effect, the surface roughness exhibits a rapid decrease within a polishing duration of 5 min, achieving a reduction of approximately 80%. Compared with the results of shear-thickening polishing in the reference literature [19], we found that there is a slight difference in the optimal range of starch concentration. This may be due to the different types and molecular structures of starch used in different experiments. And, the decrease in surface roughness is increased by about 10%. In the absence of a shear-thickening effect, the decrease in surface roughness, observed across both planar and curved interfaces, is confined to approximately 40%, indicating a limited improvement in surface smoothness. Initially, there are numerous scratches on the raw workpiece surface. The agglomerated particles due to the shear-thickening effect are able to effectively remove these irregularities. This leads to a large initial reduction in the surface roughness. Under shear-thickening enhancement for a duration of 30 min, the surface roughness is remarkably reduced. In particular, the surface roughness decreases from 3.125 μm to 0.312 μm for the flat surface (Figure 7(a-1)), from 3.098 μm to 0.353 μm for the concave surface (Figure 7(b-1)), and from 3.257 μm to 0.324 μm for the convex surface (Figure 7(c-1)). These mean improvement in the surface quality for all the workpiece geometries, exceeding those without the assistance of the shear-thickening effect. The effectiveness of this approach is evident in the significant refinement of the workpiece surfaces, underscoring its potential to improve the surface roughness accuracy and overall surface finish.

**Figure 8:**
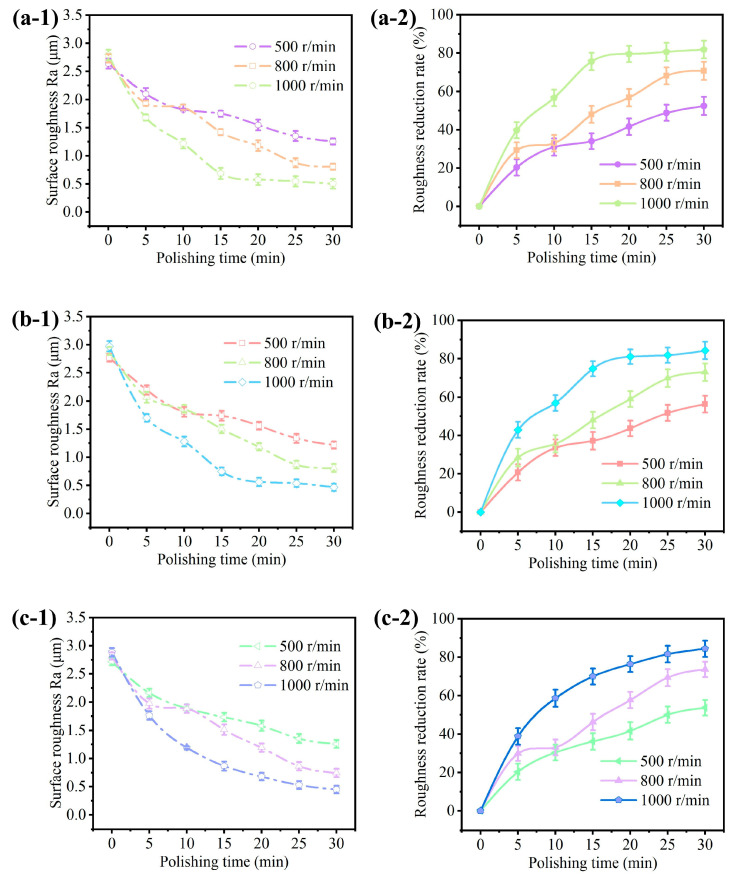
Influence of the polishing speed on the surface roughness and its reduction rate for different surface geometries: (**a**) plane surface, (**b**) concave surface, and (**c**) convex surface.

Further, we explored the influence of polishing speed on the polishing efficacy. The STF was prepared with $C_{t}$ = 5% and $C_{m}$ = 55%. The workpiece surfaces with different geometries mentioned above were polished under three rotating speeds of ω = 500 rpm, 800 rpm, and 1000 rpm at the feed depth of d = 3 mm. As shown in Figure 8, the influence of polishing speed on the surface roughness is evident. The surface roughness decreases gradually over time and ultimately tends to be stable. During the same polishing durations, the polishing speed correlates positively with the surface quality. At ω = 1000 rpm, the surface roughness is reduced to 0.512 μm after 30 min (Figure 8(a-1)), 0.508 μm (Figure 8(b-1)), and 0.517 μm (Figure 8(c-1)) for the plane, concave, and convex surfaces, respectively. When the polishing speed is halved to ω = 500 rpm, the surface roughness decreases to approximately 1.5 μm after 30 min, demonstrating poor performance compared to the condition of ω = 1000 rpm. Compared with the existing research [1], due to differences in experimental equipment, workpiece materials, and polishing fluid formulations, the specific polishing rate values may vary. Therefore, when comparing, we focus more on trends and relative changes rather than absolute values. Under the condition of doubling the polishing speed, the rate of roughness value decrease has also doubled, and the final accuracy achieved is basically the same. As the polishing speed increases, the fiber brush is hard to restore to its original state quickly, thereby diminishing the adhesion of fibers to the slurry. This reduction in grip expedites the release of abrasive particles from the polishing zone. The decreased adhesion accelerates the release of abrasive particles from the polishing zone. In addition, when abrasive particles are tightly enclosed in a colloidal particle matrix, their cutting efficiency is significantly improved. This means that the number of abrasive particles participating in the polishing process increases sharply. Therefore, the improvement of surface quality originates from the rapid elimination of more roughness peaks in the same polishing time.

**Figure 9:**
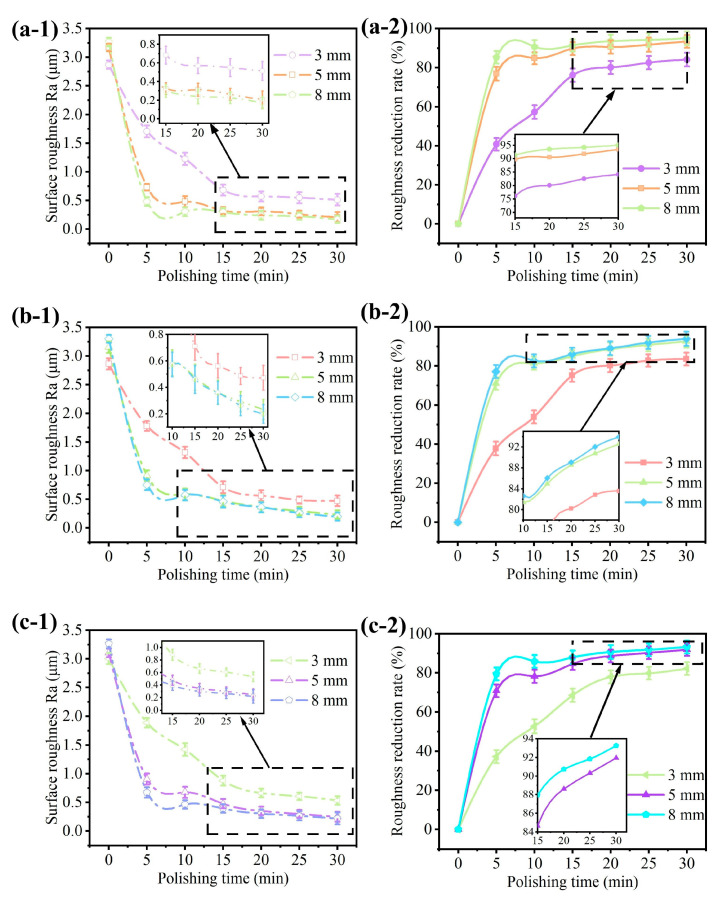
Influence of the feed depth on the surface roughness and its reduction rate for different surface geometries: (**a**) plane surface, (**b**) concave surface, and (**c**) convex surface.

When the STF is applied to polish the workpiece surface in a certain range of shear rate, the shear-thickening effect causes a significant increase in pressure on the surface. In addition, the surface pressure on the workpiece is also related to the distance between the brush fibers and the workpiece. To explore the influence of the feed depth on the polishing quality, polishing experiments were conducted for serval feed depths, d = 3 mm, 5 mm, and 8 mm at ω = 1000 rpm. The STF was prepared with $C_{t}$ = 5% and $C_{m}$ = 55%. As shown in Figure 9, the surface roughness decreases with polishing time for all the feed depths. In the first 5 min, the reduction rate was significantly higher. The reduction rate amount approached 80% at d = 5 mm and 8 mm, and 40% for d = 3 mm. After 30 min, the surface roughness further decreased. At d = 5 mm and 8 mm, the surface roughness reduced from 3.2 μm to about 0.3 μm, and most surface irregularities (such as pits and scratches) were almost eliminated. Similarly to the existing research [13], the feed depth had a significant impact on the polishing process, especially in the initial stage of polishing, where the roughness reduction rate differed by twice between the different feed depths. There was a significant improvement compared with the surface roughness of 0.873 μm at d = 3 mm. This is because the increased feed depth, which enhances the deformation of flexible fibers, improves the hydrodynamic pressure within the polishing area, consequently amplifying the polishing pressure.

**Figure 10:**
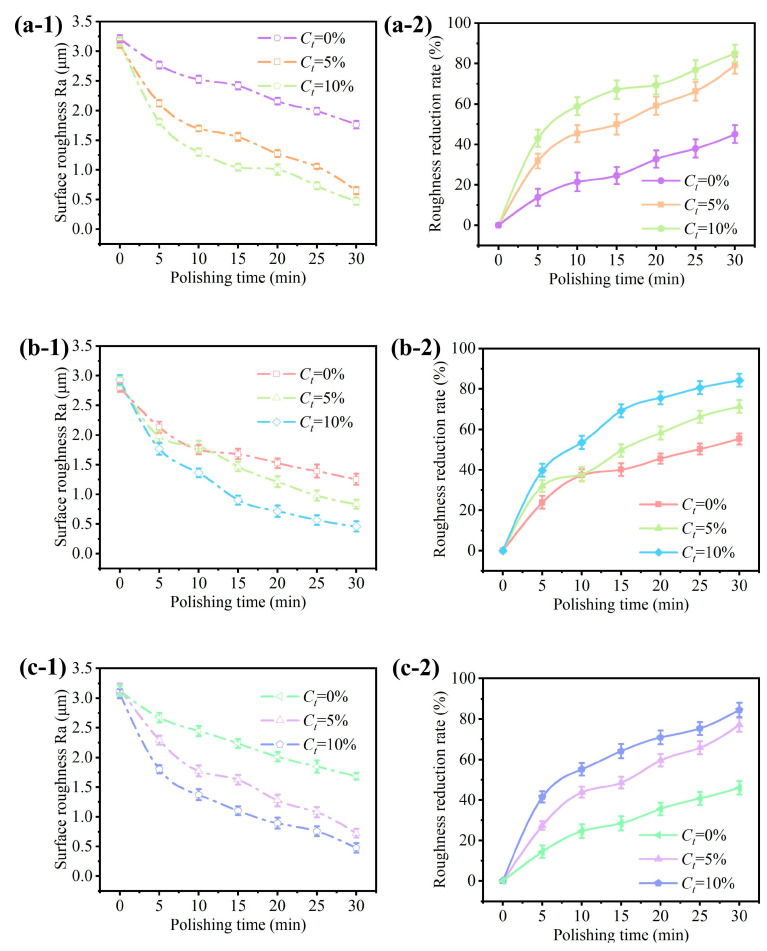
Influence of the abrasive concentration on the surface roughness and its reduction rate for different surface geometries: (**a**) plane surface, (**b**) concave surface, and (**c**) convex surface.

Figure 10 presents the evolution profile of surface roughness for different abrasive concentrations at ω = 1000 rpm and d = 3 mm. The STF slurry with $C_{m}$ = 55% was prepared at different abrasive concentrations, $C_{t}$ = 0%, 5%, and 10%. Within 30 min, the surface roughness showed an overall decreasing trend. The increase in the abrasive concentration led to improved polishing accuracy. Specifically, at $C_{t}$ = 10% the reduction rate of surface roughness exceeded 40% within 5 min, and the surface roughness during the polishing of 30 min reached about 0.5 μm, which was significantly higher than $C_{t}$ = 5% and without abrasive particles. Compared with the reference [22], adding abrasive particles to the polishing solution significantly accelerated the material removal rate, increasing it by about 50%. And, as the concentration of abrasive particles increased, the removal rate also accelerated, and the surface accuracy that could be achieved also improved. The increase in abrasive concentration improves the participation of abrasive particles in the polishing process and promotes the formation of more particle clusters under high shear conditions. In addition, increasing the abrasive concentration also helps to form denser aggregates, which enhances the polishing effect on the workpiece surface due to stronger grinding action.

**Figure 11:**
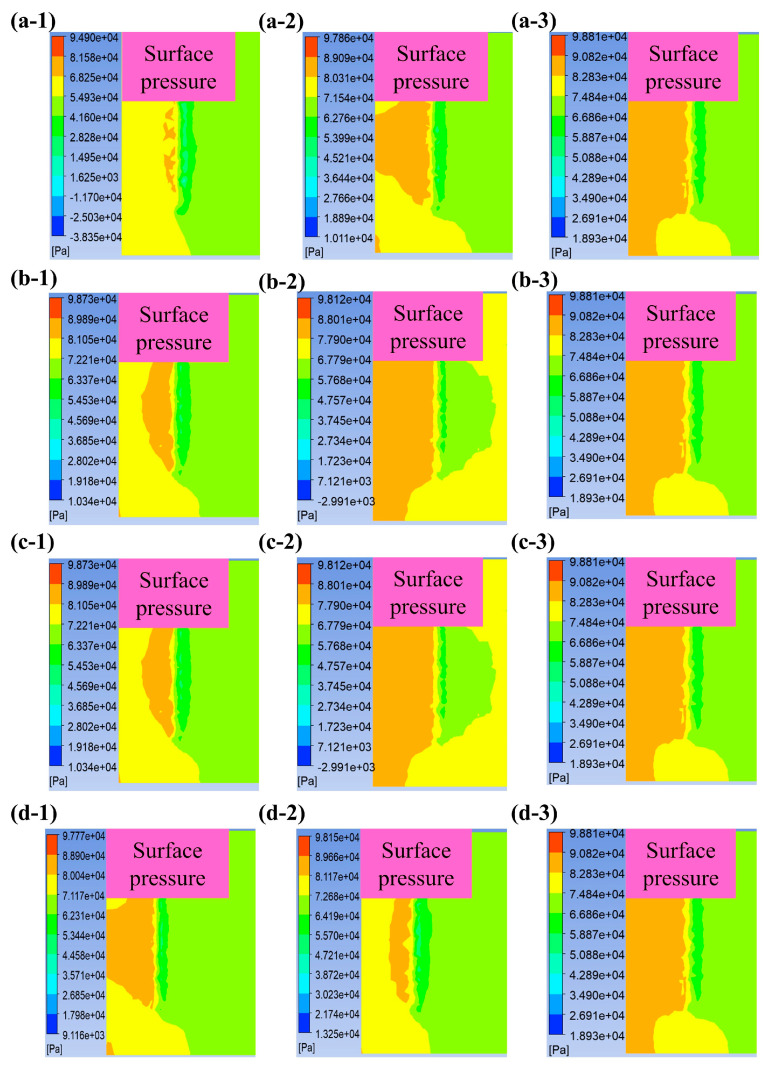
Surface pressure contours at different starch concentrations: (**a-1**) 45%, (**a-2**) 50%, and (**a-3**) 55%; at different polishing speeds: (**b-1**) 500 rpm, (**b-2**) 800 rpm, and (**b-3**) 1000 rpm; at different feed depths: (**c-1**) 3 mm, (**c-2**) 5 mm, and (**c-3**) 8 mm; and at different abrasive concentrations: (**d-1**) grainless, (**d-2**) 5%, and (**d-3**) 10%.

Numerical simulations were carried out to calculate the pressure distribution on the workpiece surface under different parameters. As shown in Figure 11, the increase in cassava starch concentration, abrasive particle concentration, polishing speed, and feed depth collectively amplify the pressure exerted on the workpiece surface. The Preston equation dictates that an increase in the surface pressure is beneficial for improving material removal efficiency. The workpiece near the brush experiences higher surface pressure (Figure 11(a-1–d-1)). The surface pressure also increases with the starch concentration (Figure 11a). At $C_{m}$ = 55%, the shear-thickening effect occurs accompanied by the formation of particle clusters, which causes the pressure to increase near the workpiece surface. As the polishing speed increases, the impact force of the slurry on the workpiece surface becomes larger, resulting in increased pressure (Figure 11b). Under the holding force of the fiber brush, the contact area between the slurry and the workpiece surface increases with the feed depth, which thus improves the surface pressure (Figure 11c). In the experiments, further increasing the feed depth at d > 5 mm has little effect on the surface roughness. The simulation results also show that there is almost no difference in the surface pressure between d = 5 mm and 8 mm (Figure 11(c-2,c-3)). The number of abrasive particles in contact with the workpiece surface per unit area increases with the abrasive concentration (Figure 11d). Therefore, there are more abrasive particles simultaneously acting on the same polishing area, which exerts stronger pressure on the workpiece surface. These are in good agreement with the experimental results.

**Figure 12:**
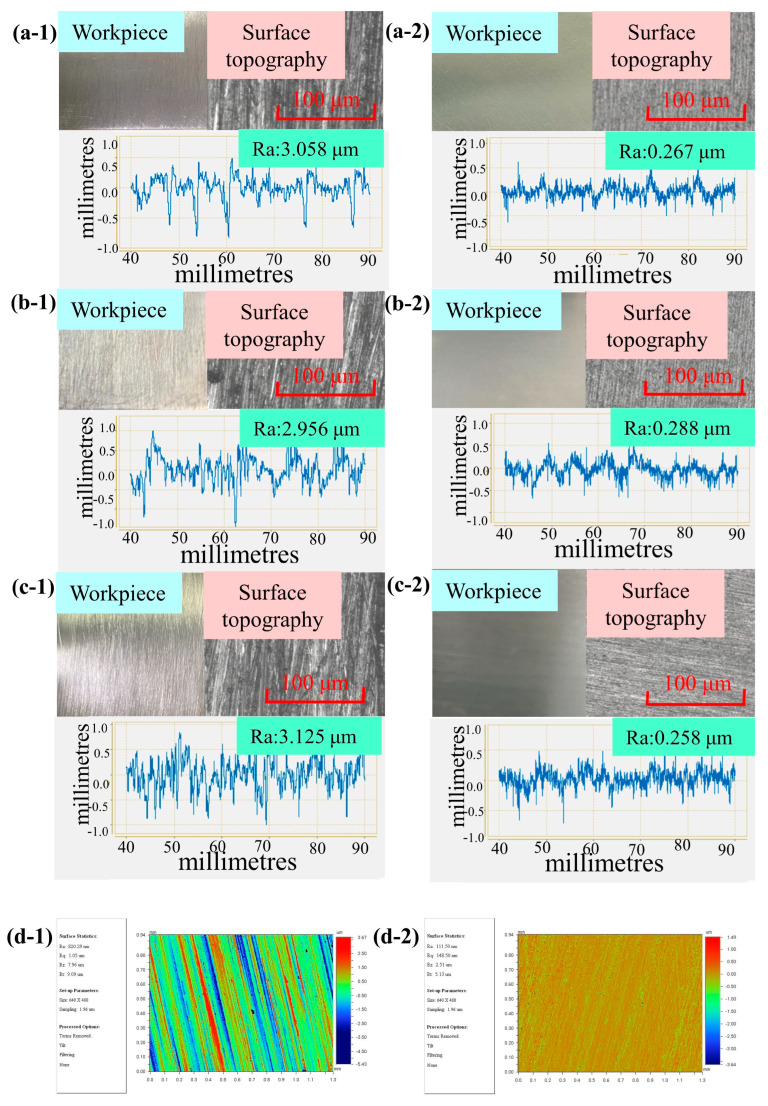
Surface topography (**left**) before and (**right**) after polishing for different surface geometries: (**a**) plane surface, (**b**) concave surface, (**c**) convex surface $(C_{t}$ = 10%, $C_{m}$ = 55%, ω = 1000 rpm, and d = 8 mm), and (**d**) two-dimensional micro-morphologies of workpiece surfaces (**d-1**) before and (**d-2**) after polishing under experimental conditions: $C_{t}$ = 10%, $C_{m}$ = 55%, ω = 1000 rpm, and d = 8 mm.

Finally, we compared the surface morphologies before and after polishing for the plane, concave, and convex surfaces. The measurement results are shown in Figure 12. The STF with $C_{m}$ = 55% and $C_{t}$ = 10% was used to polish different surface geometries of workpieces. The polishing process was carried out at d = 8 mm and ω = 1000 rpm. It was observed that the surface scratches and pits after polishing were significantly reduced. The surface roughness decreased from 3.058 μm to 0.267 μm for the plane surface, from 2.956 μm to 0.288 μm for the concave surface, and from 3.125 μm to 0.258 μm for the convex surface. Figure 12(d-1,d-2) present the two-dimensional micro-morphologies in Figure 12(a-1,a-2). Due to the short sampling distance, when we remeasured at the same surface position, the surface roughness significantly decreased from 820.29 nm to 111.50 nm. This indicates a substantial improvement in the surface morphology after polishing.

We have found that traditional polishing methods, such as mechanical polishing [15] and chemical polishing [16], typically rely on mechanical friction or chemical reactions between abrasive particles and the workpiece surface to remove surface materials. These methods often require a longer polishing time to achieve the desired surface quality. Especially for hard or complex shaped workpieces, traditional polishing methods may face problems of uneven polishing and low efficiency. STF composite materials combine the fluidity of fluids and the shear strength of solids, providing uniform polishing pressure and abrasive distribution during the polishing process. Meanwhile, the experimental results showed that compared with traditional polishing methods, the polishing time using STF composite materials was reduced by about 30%. Traditional polishing methods may also lead to surface scratches, micro-cracks, or uneven surface roughness, but the fine abrasive particles and uniform polishing pressure of STF composite materials can form smooth and uniform microstructures on the surface of the workpiece. Compared with traditional polishing methods, using STF composite materials for polishing can shorten the polishing time by 30%, reduce the surface roughness of the workpiece by more than 50%, and achieve higher surface quality standards.

## 4. Conclusions

In this work, a novel fiber brush–STF composite is prepared to implement an efficient polishing process for different surface geometries. The fiber-reinforced shear-thickening composite exhibits pronounced efficacy in refining the surface finish of polished workpieces, leading to a substantial improvement in surface roughness. The obvious shear-thickening effect emerges at $C_{m}$ = 55%. The incorporation of $C_{t}$ = 5% abrasive into the STF results in a maximum viscosity of approximately 4.27 Pa·s, while at $C_{t}$ = 10%, the viscosity reaches roughly 9.81 Pa·s. It implies that enhanced particle–particle adhesion prompts viscosity augmentation. The effects of different properties of the composites on the surface roughness Ra of workpieces are systematically analyzed. The findings indicate a remarkable positive correlation of the surface roughness with the polishing speed, abrasive particle concentration, and feed depth. It was found that under reasonable parameters, the roughness value can reach a minimum of 0.14 μm, accompanied by the efficient elimination of surface imperfections at the processed zone. These results serve as a reference value for optimizing polishing strategies aimed at refining the surface quality of complex curved geometries. Meanwhile, the simulation results show that compared to deionized water as the polishing solution, the pressure on the workpiece surface is increased when the STF composite is used as the polishing solution. According to the Preston equation, an increase in the polishing pressure can improve the material removal rate, which is consistent with the experimental results. In the paper, process parameters such as polishing speed and abrasive mass fraction have a significant impact on the polishing effect. However, the current research on optimizing these parameters is not sufficient, and there is a lack of theoretical models to guide the selection and optimization of process parameters. In the future, we can deepen our research on theoretical models to more accurately guide the selection of process parameters.

## Figures and Tables

**Table 1:** Experimental conditions.

Experimental Parameters	Value
Abrasive	SiC
Base-fluid	Cassava starch and water
Abrasive size	13 μm
Mass fraction of cassava starch	45%, 50%, 55%
Slurry temperature	10 °C, 25 °C, 40 °C, 65 °C

**Table 2:** Experimental parameters.

Parameters	Value
Cassava starch concentration	45%, 50%, 55%
Motor speed (rpm)	500, 800, 1000
Feed depth (mm)	3, 5, 8
Concentration of SiC	5%, 10%, grainless
Cassava starch concentration	45%, 50%, 55%

## Data Availability

The original contributions presented in this study are included in the article. Further inquiries can be directed to the corresponding author.

## Appendix A

The flow simulations of the polishing on the workpiece surfaces were carried out through solving the conservation equations of mass and momentum. The continuity equation is expressed as follows:

(A1) $$\frac{\partial \rho }{\partial t}+D\cdot \rho u=0,$$

where ρ is the fluid density, and u is the fluid velocity. The conservation equation of momentum is given as follows:

(A2) $$\rho \frac{\partial u}{\partial t}+(u\cdot \nabla )u=-\nabla p+\mu \nabla^{2}u+f,$$

where p is the pressure in the fluid, *μ* is the fluid viscosity, and f is the external volumetric force acting on the fluid. The viscosity constitutive equation for non-Newtonian power-law fluids was used for the simulations, with the values of *K* and *n* from Section 2.2:

(A3) $$T=K{\left(\frac{\mathrm{d}\nu }{\mathrm{d}x}\right)}^{n},$$

The dimensions of a single fiber bristle are set to Φ 0.32 × 24.50 mm, operating at a rotational speed of 1000 rpm. To improve computational efficiency and accuracy, we used a mesh refinement strategy on the local regions near the fiber bristle and the workpiece. The time step is set to 0.002 s, along with an iteration limit of 200 steps. The fluid/solid coupling simulations are iteratively executed until the prescribed convergence is reached for individual time steps.
